# The American and Colonial Journals: Midwifery, and Diseases of Women and Children

**Published:** 1842-10

**Authors:** 


### MIDWIFERY, AND DISEASES OF WOMEN AND CHILDREN.

### *Atresia Vaginæ.* By Dr. MARSH, of Delaware.

A young woman, aged 18, complained of symptoms indicating dysmenorrhea. On inquiry, it was found that she had menstruated at a very early period, and had continued to do so regularly up till a few months previously. Examining per vaginam, the author discovered a strong septum, no doubt the result of inflammation, occluding the passage to the womb. This was divided, and the operation was followed by a jet of tar-like blood, which spouted to the height of one or two feet, and flowed to the extent of five or six pounds. The relief was complete.

[A nearly similar case is given in No. 5 (Jan. 29, 1842) of the same Journal. In this case, the existence of the septum, which was not complete, a small central part being patent, was only discovered during parturition. The practitioner resolved to leave the membrane untouched, "in order that he might observe how far the unaided efforts of nature would overcome the obstruction," and knowing that he "could at any time divide it." The membrane dilated, and that without rupture, sufficiently to allow the birth to take place: but the child came into the world asphyxiated, and was only recovered after great exertions. The practitioner's conduct was plainly reprehensible.]

*Medical Examiner.* No. i. *Jan.* 1, 1842.

